# Managing health disasters and Civil–Military Cooperation: A case of COVID-19 in Pakistan

**DOI:** 10.4102/jamba.v13i1.1113

**Published:** 2021-08-24

**Authors:** Rabeea Jabbar, Muhammad Makki

**Affiliations:** 1 Centre of International Peace and Stability, Faculty of Social Sciences, National University of Sciences and Technology, Islamabad, Pakistan

**Keywords:** natural hazards, health disasters, pandemic, CIMIC, disaster management, COVID-19, disaster medicine, Pakistan

## Abstract

As an institution, the Pakistan Army has been playing a significant role in dealing with emergencies and disasters facing the nation. The novel coronavirus disease, Severe Acute Respiratory Syndrome (SARS) COVID-19, was wreaking havoc around the world. The pandemic is a threat to health and has caused severe damage across most aspects of the society. The situation forced the formation of a unique series of civil–military inter-agency relationships to be formed, in order to curb the spread of the pandemic. The Pakistan Army that was neither trained nor equipped to undertake any such health disaster management operation played a significant role in preventing disease and overcoming the disaster. Civil–military cooperation (CIMIC) was the key to the successful response of Pakistan towards COVID-19. The research was based on qualitative interviews that analysed the phenomenon of COVID-19 pandemic, that is, ‘health disaster’, to elucidate the disaster management practices performed through the framework of CIMIC in Pakistan. With regard to this, the article argued that formulating a comprehensive guideline or framework was necessary to maintain an effective and cooperative relationship between civil and military components. It further demonstrated the need to recognise the constitutive factors that influenced the functionalisation and institutionalisation of CIMIC to manage the highly complex health-related emergencies.

## Introduction

Civil–military cooperation (CIMIC) has become a catch-phrase in the disaster and crises management agendas of the 21st century, considering the unprecedented nature of the emerging natural disasters (AghaKouchak et al. 2018; Zhou et al. 2018). In particular, the Asia-Pacific region has been experiencing intensified and complex natural disasters comparable to any other region (Alisjahbana, Zahedi & Bonapace 2019; Price 2019). In a similar vein, Ma and colleagues (2016) argued that the emergence of novel and re-emergence of some old infectious diseases have exposed several countries to health emergency and disaster risk management (DRM) challenges. Climate change, rapid urbanisation and declining ecosystems (Chabas et al. 2018; Smith & Fazil 2019) are amongst the most frequent involved factors in the rise of (new) infectious diseases (Cuthbertson et al. 2019). As the nature of the health disasters has changed, the need for a synergised intra-institutional response has also increased, especially regarding ‘civil’ and ‘military’ cooperation (i.e. CIMIC). The application of CIMIC whilst dealing with health crises is not a new phenomenon, but instead there is a long pedigree of military involvement (as an institution) in health emergencies (Licina 2012; Michaud et al. 2019).

In late December 2019, the first official coronavirus disease (COVID-19) cases were recorded in China. At the beginning of the outbreak, no one could have predicted its far-reaching impacts. However, the disease subsequently spread to almost all the countries worldwide, infecting more than 29 million people, causing deaths of more than 900 000 (WHO 2020). Hence, the COVID-19 pandemic proved to be a unique humanitarian and public health crisis (Dong & Bouey 2020; Topcuoglu 2020). The pandemic did not only confine itself as a mere health emergency or crisis but also impacted socio-economic, political and security aspects (Bricknell 2021; McKibbin & Fernando 2020).

Similarly, in Pakistan, the first case of COVID-19 was reported on 26th February 2020 (Abid et al. 2020; Waris et al. 2020). After a brief hiatus following the first case, there was a sharp spike in the number of cases. Several commentators reported that most infected patients included pilgrims who travelled to Iran^1^ (e.g. Ellis-Petersen & Baloch 2020; Mandhro 2021). This increase in the number of COVID-19 cases resulted in the partial lockdown situation all over the country.

On the account of facts stated above, the worsened situation across the globe necessitated the functionalisation of CIMIC in order to mobilise cross-institutional capacities or the resources to effectively counter and manage the crisis (Bricknell 2021; EUROMIL 2020). Therefore, it is essential to understand the complex dynamics surrounding CIMIC with regard to crisis management infrastructure. Pakistan also mobilised its military to assist and coordinate with civilian authorities in dealing with the pandemic (Gul 2020). Here, it is important to recognise that CIMIC has been well-exercised in Pakistan during various natural disasters. Whilst considering the institutional resources and capacity, the Pakistan military has been observed as the first respondent to any crisis in Pakistan, be it floods or earthquakes (Raza & Kandhro 2015; Salil 2012). Considering the unique nature of the health crisis in the shape of COVID-19, the Government of Pakistan once again effectively managed the ‘risk’ through an institutionalised mechanism: that is the establishment of the National Command Operation Centre (NCOC), an apex coordinating body between ‘civil’ and ‘military’ authorities (Faisal 2021).

There has been considerable interest amongst researchers in understanding the role of CIMIC in health disaster management (e.g. Opillard, Palle & Michelis 2020; Sari 2020). However, there is a greater need to explore the dynamics of CIMIC in Pakistan in a health emergency or health-crisis situation. Therefore, this article aims to understand the disaster management practices related to CIMIC in Pakistan, focusing on the case of the COVID-19 breakout. It then highlights the civilian military – cooperative emergency response mechanisms to infectious diseases (such as COVID-19), the joint working and disposal mechanism to prevent and manage the health disaster in Pakistan. This article further highlights the need to formulate a guideline and establish a model framework to maintain practical cooperation between military and civil authorities through an institutionalised policy response.

## Civil–military cooperation, health emergency and disaster (risk) management

The World Health Organization (WHO) defines a ‘disaster’ as a phenomenon that causes severe disruption of society’s functioning and causes human, material, economic and environmental damage on a large scale (WHO 2014). Scholars have categorised disasters into two main types: natural disasters and human-made disasters. The global frequency of natural disasters ‒ such as floods, droughts, landslides, avalanches, cyclones, smog and epidemics–pandemics–endemics have increased over the past few decades as a result of climate change because of rapid industrialisation processes (Padli, Habibullah & Baharom 2018; Thomas & López 2015). The institutions engaged in disaster-related research have identified the emergence of new and unprecedented health disasters and re-emergence of some old disastrous infectious diseases because of underlying conditions such as poverty, climate change, rapid urbanisation, declining ecosystems, etc. (Cuthbertson et al. 2019; ed. Veenema 2019). Prior research suggests that these public health emergencies pose a severe threat to humanity and can result in complex disasters if not dealt with appropriately (Alwidyan, Trainor & Bissell 2020).

**FIGURE 1:**
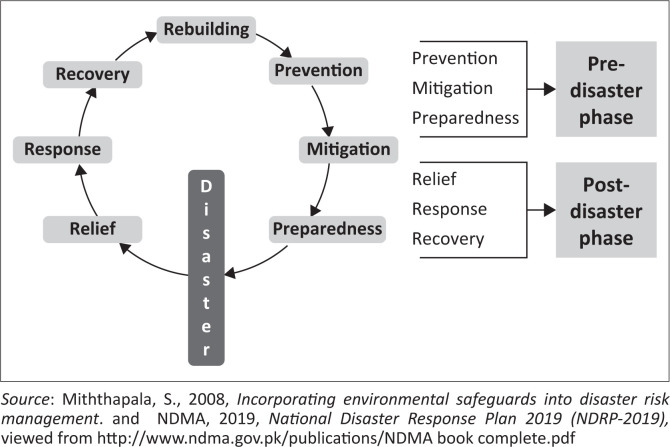
Six phases of disaster management cycle.

In the last few years, however, we have witnessed that public health has become a sub-specialty area of disaster management and is now known as ‘disaster medicine’ (Burkle Jr. 2019). There are six phases involved in the disaster management cycle (see Figure 1). It has been observed that disaster medicine has modified classic Carr’s Cycle of Disaster Management and has introduced its own disaster cycle, which comprises all the actions taken before, during and after the event to fit in the area of public health (e.g. Khan et al. 2008). Despite all this, Burkle (2016) observed that, during the last few years, the disaster management authorities remained significantly invested in the disaster response phase that led to increased morbidity and mortality rate in health crises that could have been prevented. Here, disaster management’s preventive aspect has remained one of the key concerns (Burkle Jr. 2019; Randolph, Chacko & Morsch 2019; Veenema 2019).

Jester, Uyeki and Jernigan (2018) argued that to ensure effective management of pandemics, the disaster cycle’s prevention and preparedness phases need to be well-considered. More importantly, such prevention and preparedness phases require considerable CIMIC to optimise localised response. Indeed, the interaction between ‘civil’ and ‘military’ authorities may create a contested zone. However, their cooperation in an integrative manner is essential to manifest effective disaster management (Licina, Burkle & Kamradt Scott 2016; Watterson & Kamradt-Scott 2016). Consequently, we have observed a long pedigree of necessitated cooperation between civilian and military authorities in fights against the infectious diseases (such as the fight against yellow fever, malaria and the introduction of tropical medicines) (Araghizadeh et al. 2020; Licina 2012; Michaud et al. 2019; Wenham 2019). In fact, militaries form an essential part of the disaster management infrastructure in most countries (e.g. see Bricknell 2021).

From the outbreak of Avian Influenza in China to the epidemics of Ebola in the African continent and SARS and Middle East Respiratory Syndrome (MERS) in China and South-East Asia, we have witnessed effective prevention and management of infectious diseases (i.e. health emergencies) through CIMIC mobilisation (via clusters of involved sub-organisations) (Michaud et al. 2019; Ma et al. 2016). This programmed approach has provided states with yet another opportunity to enhance CIMIC in a wide range of areas, such as post-conflict reconstruction, rehabilitation, counter-terrorism, research cooperation, etc. (e.g. Licina, Burkle & Kamradt Scott 2019; Ma et al. 2016; Watterson & Kamradt-Schott 2015). Rosén (2009) believed that we are witnessing a third generation of CIMIC where the involved complex web of civil and military components are observing new patterns of relationship (Brooks 2019; Cook & Yogendran 2020).

## Methodology

To address the research inquiry, a qualitative approach was adopted. This study was conducted over a period of 6 months, that is, from April 2020 to September 2020. A total of 12 in-depth and semi-structured interviews were conducted with experts (both military and civilian authorities) in the fields of crises and disaster management. An open-ended (thematically driven) interview protocol was used and consisted of 15 questions covering a broad range of topics such as the functional dynamics around the establishment of the NCOC and the National Disaster Management Authority (NDMA) in Pakistan, civil and military coordination in (health) disaster management and how an institutionalised approach can strengthen the human security agenda at the national level. Particular attention was paid to recruiting respondents who are currently working in the COVID-19 scenario or have had similar past experiences in health disaster management. The interviews were telephonically conducted and recorded with the respondents’ permissions. It allowed gathering more detailed explanations in the aforementioned areas (e.g. Smith 2005).

To ensure research objectivity, transparency and compliance with accepted principles, this research received ethical approval from the Centre for International Peace and Stability (CIPS). The confidentiality aspect was also considered when few respondents preferred their identities not to be disclosed. Furthermore, required official approvals were secured from the relevant departments before conducting the interviews. Before the formal interview sessions, the respondents were provided with a project information sheet and informed consent form, containing detailed information regarding the overall research objectives.

Thematic analysis of the interviews was carried out in five stages. In the first step, the recorded interviews were transcribed. In the second step, the authors were familiarised with the data by reading it carefully whilst keeping the theoretical lens in consideration. In the third step, the data were coded in relation to major themes surrounding CIMIC during COVID-19. The fourth step identified different themes in the data, which were clustered around the research objectives. In the last step, the data were theoretically and conceptually compared with the relevant literature to establish coherency between the gathered data and existing literature. Regarding the CIMIC during COVID-19 crisis in Pakistan, four main themes emerged: (1) the significance of CIMIC in disaster management, (2) challenges associated with CIMIC during the COVID-19 pandemic and (3) the potential role of NCOC. In addition, various government policies and practices related to natural hazards and disaster management proved to be of significant help in understanding the dynamics of CIMIC (with regard to disaster management).

## Data: The evolution of Pakistan’s national disaster management system

Pakistan is located in South Asia and shares borders with China in the Northeast, Afghanistan in the West and Northwest, Iran in the Southwest, the Arabian Sea in the South and India in the East. Having a diverse landscape varying from mountains to plains, deserts, forest hills and plateaus and being home to a few of the tallest and longest mountain ranges in the world, that is, the Karakorum, Himalayan and Hindu Kush, the country lies in the extremely active seismic zone and its vulnerability to the natural disasters could be ranked from moderate to severe on the scale (Bacha 2016; NDMA 2019). Therefore, because of its unique geographical location and diverse climatic conditions, the country is prone to natural and human-made disasters. Earthquakes, floods, landslides, cyclones, droughts, etc., have been frequent phenomena, particularly in the last two decades (see Table 1).

**TABLE 1:** Summary of damages and losses in years (2007‒2017).

Disasters	Losses and damages		
	Deaths	Injuries	Houses damaged
Floods 2017	271	359	996
Floods 2016	153	113	1452
Pre-Monsoon Heavy Rains 2016	271	279	2929
Floods 2015	238	232	10 716
KP Earthquake	272	856	96 152
Floods	367	673	107 152
KP Tornado	49	267	-
Floods 2013	333	173	79 943
Awaran Earthquake 2013	386	816	46 756
Mashkel Earthquake 2013	14	52	2000
Floods 2012	571	2902	636 438
Floods 2011	520	1180	1 604 406
Super Floods 2010	1985	2946	1 602 765
Ziarat Earthquake	164	173	9761
Floods 2008	80	21	17 721
Cyclone Yemyin 2007	443	-	71 486

*Source*: NDMA, 2019, National Disaster Response Plan 2019 (NDRP-2019), viewed from http://www.ndma.gov.pk/publications/NDMAbookcomplete.pdf

As mentioned earlier, Pakistan is a host to various natural and human-made disasters because of its topographic and demographic situation. Some of them are seasonal and occur annually, whereas earthquakes, tsunamis and other such disasters occur rarely but cause severe infrastructural and economic damage to the country. However, an increase in the frequency and intensity of these rare hydro-meteorological disasters induced by climate change can be observed since the early 2000s (Glasser 2019). Therefore, a need for adopting a Disaster Risk Reduction (DRR)/Management policy along with capacity building measures was realised and the country’s first-ever National Disaster Plan was formulated and adopted in 2010. Before the promulgation of *the Disaster Management Act 2010*, Pakistan’s disaster management approach was ‘responsive’ in nature and was solely focused on the post-disaster phase.

**FIGURE 2:**
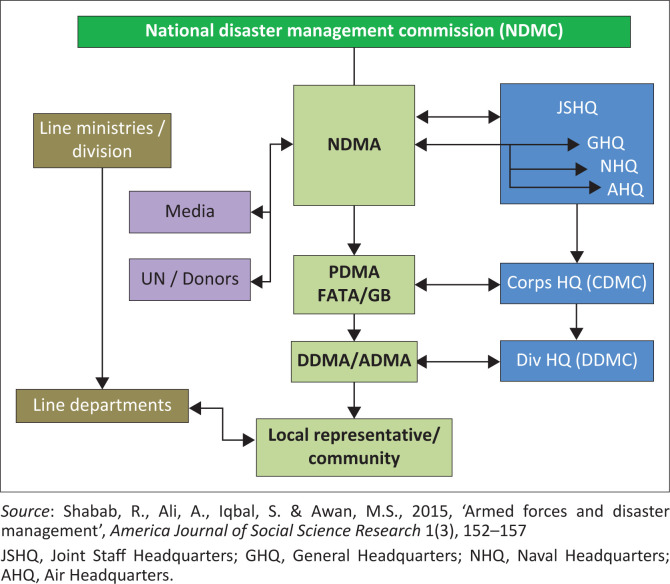
National disaster management layout: Key players/stakeholders.

Nevertheless, a paradigmatic shift can be observed in this approach as Pakistan experienced consecutive disasters of massive magnitude during 2010, 2011 and 2012 (Shabab et al. 2015). In order to deal with such immense disasters, under the *NDM Act of 2010*, a three-tiered disaster management regime was adopted with NDMA at the Federal level and Provincial Disaster Management Authorities (PDMAs) and District Disaster Management Authorities (DDMAs) at provincial and district level, respectively (Bacha 2016). See Figure 2 for the detailed National Disaster Management Layout of Pakistan.

Owing to international frameworks such as ‘Sendai Framework Agreement 2015’, ‘Sustainable Development Global Framework 2015’ and ‘Paris Agreement 2015’, Pakistan has adopted many DRR-related programs. Also, the recurrent disasters after 2010, such as earthquakes of 2013 and 2015, floods (during 2010, 2011, 2012, 2014, 2015, 2016 and 2017) and Yeymin-Phet cyclones, the Government of Pakistan revised its ‘National Disaster Response Plan’ in 2019. The primary objective was to improve the country’s disaster management regime using a comprehensive national approach. The approach is often titled as ‘Multi-hazard Response Plan’ (NDMA 2019).

The military of Pakistan has been at the forefront with regard to disaster response(s). Under Pakistan’s constitution, the Armed Forces of Pakistan are responsible for assisting the civilian authorities in any emergency. Hence, both the National Disaster Response Plan of 2010 and its revised version of 2019 specify he Army’s role as ‘supportive’ in nature. However, it is crucial to observe that NDMA identifies the Army’s role in the ‘post-disaster phase’ that involves relief and rescue operations and the management of internally displaced persons (IDPs) and the provision of security. Therefore, although the military is an important actor in disaster relief activities, it has a secondary role in disaster management (Raza & Haq 2015).

### COVID-19 and civil–military cooperation: The case of Pakistan

Over 295 053 have reportedly been infected in Pakistan during the global COVID-19 pandemic (Government of Pakistan 2020). Since the first case detected in late February, more than 6000 people have lost their lives fighting against the deadly disease, whilst the disease has spread to almost all parts of the country. The highest cases have been reported in Sindh, followed by Punjab and Khyber Pakhtunkhwa (Khan, Saeed & Ali 2020; Government of Pakistan 2020). For the province-wise confirmed COVID-19 cases in Pakistan, see Table 2.

**TABLE 2:** Province-wise COVID-19 confirmed cases in Pakistan.

Variable	Confirmed cases	Active cases	Deaths	Recoveries
Azad Jammu & Kashmir	2277	114	61	2102
Balochistan	12 742	965	141	11 636
Gilgit-Baltistan	2816	337	67	2412
Islamabad	15 578	480	175	14 923
Khyber Pakhtunkhwa	35 923	964	1250	33 709
Punjab	96 636	2020	2195	92 421
Sindh	129 081	3953	2394	122 734

*Source*: Government of Pakistan, 2020, COVID-19 Health Advisory Platform by Ministry of National Health Services Regulations and Coordination, viewed 21 June 2020, from https://covid.gov.pk/stats/pakistan

With the weak public health infrastructure and lack of emergency preparedness mechanisms (Pakistan spends less than 1% of its total GDP on public healthcare annually, see Ullah et al. 2020), Pakistan found itself highly exposed to COVID-19 infiltration from neighbouring China and Iran. Consequently, when the World Health Organization (WHO) declared the COVID-19 a ‘global health emergency’, the Government of Pakistan had no choice but to use all its (financial and institutional) resources in preparing for what was coming. Several respondents pointed towards the absence of a health plan or policy that is well-equipped to manage health crises such as COVID-19 (e.g. pers. comm., 10 August 2020).

Consequently, the Government of Pakistan formulated a National Action Plan (NAP) that served as a blueprint in managing and preventing the health and socio-economic impacts of COVID-19. The NAP was formulated in light of the information provided by both WHO and the Chinese government to ensure a coherent response at the global stage.

Several respondents acknowledged the unprecedented nature of COVID-19 crisis that led to a unique collaboration between civilian and military authorities. The response became unmanageable as there was a spike in the number of cases because of pilgrims’ return from Taftan, Iran (Ellis-Petersen & Baloch 2020; Mandhro 2021). No civilian disaster management agency felt fully prepared to deal with the contagion because of scarcity of resources, training and infrastructure. The civilian organisations felt overwhelmed, especially as death rates continued to climb. In this vein, a senior military official believed that the crisis tested the efficacy and institutional strengths (pers. comm., senior military official, 19 June 2020).

Therefore, Pakistan’s healthcare system and disaster management system were indeed not ready to deal with the looming crisis when it first started. There were not enough ventilators and isolation wards and even personal protective equipment (PPEs) for the doctors and other healthcare staff were in significant shortage. In addition, the country’s testing capacity was highly deficient and there was no way to follow the 3T policy (Trace, Test and Treat) (Yilmaz & Aydin 2020).

Two further issues compounded the situation. Firstly, the NDMA (as an organisation) was essentially formed to tackle the traditional natural disasters such as earthquakes, floods, etc. It was not adept at responding to challenges posed by a pandemic such as COVID-19. Secondly, there was a grave lack of coherence between the national and provincial governments, and other concerned authorities.

As a result, considering the vast experience of the Pakistan military and its institutional capacity or resources in disaster relief operations, the military was to assist the civilian authorities in dealing with the COVID-led situation:

Disasters and calamities are a part of the routine, particularly in countries with weak governance systems. Pakistan’s armed forces since the inception of Pakistan, armed forces of Pakistan have always been at the forefront of disaster-related activities, be it floods, earthquakes, or any other natural disaster. Their familiarity and experience with such situations along with their institutional strength, led to the establishment of the National Command and Control Center (NCOC). (pers. comm., military official, NCOC, 06 August 2020)

**FIGURE 3:**
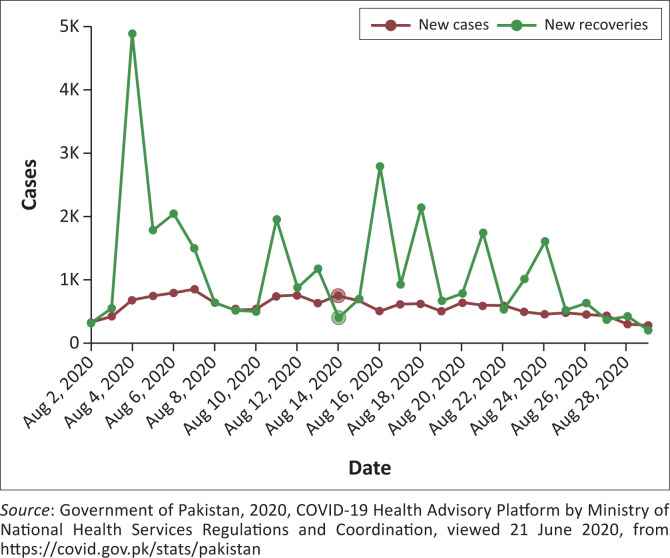
Graphical representation of ratio of new COVID-19 cases versus the rate of recovering patients.

Therefore, cooperation between civilian and military authorities to manage the health disaster was necessary. The role of the Pakistani military is well-specified in the constitution of Pakistan and under the ‘National Response Plan of 2010’. However, it is important to notice that the military has only been engaged in ‘relief operations’. Therefore, in response to the COVID-19 situation, it is the first time that a common headquarter (i.e. NCOC) for the coordination between the civil and military organisations regarding policy decision-making has also been established in the case of a health emergency. This type of collaboration between the two departments is unique in nature and has never been observed before. See Figure 3 for the new cases versus newly recovered in Pakistan.

### COVID-19, *civil–military cooperation and national command operation centre*

National Command Operation Centre officially started its functioning in April 2020. During the time period, the country had minimal capacity to manage the unprecedented growth of COVID-19. In addition, the country was facing severe socio-economic impacts, particularly following the enacted countrywide lockdowns. Nevertheless, the establishment of NCOC converged diverse actors into an ‘operational framework’ to create a synergised response (pers. comm., Ministry of Civil-Defense, Gilgit-Baltistan, 12 August 2020).

Several respondents believed that the establishment of NCOC gave a ‘direction’ to Pakistan’s response against COVID-19. For instance, a senior military official stated that the NCOC was established to enable quick decision-making through coherent response options focusing on the national economy whilst keeping the provinces on-board. This is still being carried out by enhancing inter-ministerial coordination with the help of committees that discuss the concerning issues on an almost daily basis arranged at NCOC, Rawalpindi.

Furthermore, one of the major objectives of NCOC was to enhance the COVID-19 testing capacity in Pakistan. With regard to this, the NCOC achieved phenomenal results: from 200 persons per day to 52 000 persons per day. Correct and timely collection of data, pooling up of authentic and updated repository of patient data, technical coordination with provincial Information Technology (IT) teams by the integration of Integrated Disease Information Management System (IDIMS) with provincial Disease Surveillance Systems (DSS) and monitoring of timely and accurate data entry are other essential milestones achieved through the platform of NCOC. National Command Operation Centre was also made responsible for formulating detailed guidelines for paramedics, industries and organisations, smart lockdown policies, identification of loopholes in the distribution of money under *Ehsaas Programme,*^2^ bringing back stranded Pakistanis from across the world and media management to create awareness amongst masses about the precautionary measures to be taken during this pandemic.

National Command Operation Centre also became an authority to develop ‘Resource Management System’ (RMS), an application developed with the collaboration of ‘National Information Technology Board’ (NITB)^3^ that provides visibility of healthcare facilities, including critical health equipment of all hospitals across the country (The Express Tribune 2020a). This application provides near real-time visibility of all COVID-19-related facilities and their source for effective planning and monitoring. The deployment of the system has been completed and can be accessed through all hospitals around the country. Baseline data compiled through the help of the Pakistan Army are uploaded through the application and continuous monitoring is being performed to ensure a regular update to establish Pakistan’s national repository. In addition, a decision support system (DSS) has also been launched to enable decision-makers to identify hotspots and clusters. It is a system that envisions an automated generation of alerts to district authorities to enable them to take reactions of masses in order to implement smart lockdowns and ensure public safety whilst showing visibility to NCOC level (pers. comm., senior military official, 19 June 2020). In addition, NCOC has also launched a helpline for the healthcare workers and enables hospital workers to lodge complaints (The Express Tribune 2020b). The helpline will also help to address structural inadequacies of the national healthcare system’ (pers. comm., former DG NDMA, 19 June 2020).

Besides the Pakistan Army’s efforts in NCOC, several respondents also pointed out the logistical support provided by the military to the civilian authorities. For instance, it was mentioned during an interview with a military official that the Army has played a pivotal role in the distribution of relief packages and critical medical equipment such as testing kits, ventilators, PPEs and medicines throughout the country (pers. comm., a senior civilian official, Military Land and Cantonment Group [MLCG], 20 June 2020). Another respondent from a civilian provincial authority shared his views as:

Despite the non-coordination and trust-deficit claims between civil and military authorities, we faced no such issue at all. The military assisted us in a highly effective manner. They [*military*] helped us isolate and quarantine the infected villages or towns. Smart lockdowns were enforced in the COVID-19 hotspots (pers. comm., MLCG, Noshki, Balochistan, 21 June 2020)

The military provided the civilians with logistical support, but they also donated a considerable sum from their salaries to corona relief funds (The Express Tribune 2020c). ‘Armed Forces Institute of Rehabilitation Medicine’ (AFIRM)^4^ that was used initially to treat persons with disabilities was converted into 130-bedded quarantine and isolation facilities. Similarly, significant portions of all Combined Military Hospitals (CMHs) in Pakistan were allocated for civilian patients. The COVID-19 crisis has indeed exerted pressure over the existing institutional capacities; however, the synergetic response through effective CIMIC has proven to be highly effective in Pakistan’s context; however, the synergetic response through effective CIMIC has proven to be highly effective in Pakistan’s context. Nevertheless, several respondents highlighted several gaps or challenges which can be broadly associated with disaster-related policy-making and management. For instance, the respondent acknowledged that the concerned disaster management-related authorities faced enormous challenges because of the dysfunctional civilian local governance. In contrast, the Pakistan military is widely considered a well-organised institution with systematically functionalised distributed power hierarchies.

In addition to the above, certain legal dynamics can also be considered as hindering the synergetic response against COVID-19. In this regard, during a conversation with a senior military officer, our attention was pointed towards the issue of 18th Amendment^5^:

In my opinion, one of the main reasons behind the establishment of NCOC, in the presence of an already functioning disaster management system, is that NDMA is a federal-level agency that shares its national jurisdiction with the provincial agencies. [*However, t*]his causes hurdles in many of the activities carried out at the federal level and also created barriers in the way of an articulated response […]. (pers. comm., military official, 19 June 2020)

In addition, several respondents, particularly military officials, raised their concerns considering the ‘only’ military role in the post-disaster phase of DRM. According to them, there is a greater need to realise the importance of civil and military involvement in preventing mechanisms.

Overall, the respondents acknowledged the unique collaboration between civil and military authorities in the COVID-19 crisis. According to them, all the organisations worked together towards a common goal, that is, to contain coronavirus. The response of the NCOC evolved on the weekly and sometimes daily basis, considering the rapidly evolving epidemiology.

## Discussion

This article elucidated the role of CIMIC in the disaster management practices of Pakistan and how the framing of COVID-19 as a ‘health disaster’ led to a unique CIMIC. We identified barriers and opportunities for CIMIC during disaster management activities in Pakistan.

In recent years, disaster-related studies have warned about the emergence of new and re-emergence of some old infectious diseases because of climate change, unhygienic conditions, deforestation, urbanisation etc., (Cuthbertson et al. 2019; ed. Veenema 2019). Scholars have highlighted that public health has become a sub-specialty area of disaster management (Burkle Jr. 2019).

Repeatedly, scholars have criticised the approach of disaster management authorities who are usually solely invested in the post-disaster phase of the DRM cycle that increases the morbidity and mortality rates during the health crises (e.g. ed. Veenema 2019).

## Conclusion

In the case of COVID-19 pandemic, the unpreparedness of the disaster managing authorities of almost all the countries (including Pakistan) has resulted in the loss of human lives that could have been prevented. Pakistan spends a meagre amount of its Gross Domestic Product (GDP) on its public healthcare and disaster management departments annually. These funds are typically utilised in ‘response and relief’ campaigns (i.e. after the disasters) and not in the ‘preparation and prevention’ phases.

Civil–military cooperation has been well-noticed by various scholars in managing the health crisis (Licina 2012; Michaud et al. 2019). This article also observed that with the establishment of NCOC and Armed Forces of Pakistan’s assistance to civilian authorities, Pakistan is effectively managing the COVID-19 crisis. The ‘civil’ and ‘military’ components have been working in coordination and coherence whilst regarding each other’s autonomy. As discussed earlier, several scholars (e.g. Kohn et al. 2010; Rosen 2009) have also argued that both the ‘civil’ and ‘military’ have started becoming less ‘dramatic’ in the third generation of CIMIC. However, this article also observed a communication gap between the ‘civil’ and ‘military’ authorities, owing to different institutional mandates and ‘chains of command’; however, they worked together towards a common goal and/or cause.

This article also highlights the lack of both national and international guidelines driving CIMIC. Therefore, formulating a guideline and establishing a framework to maintain CIMIC are necessary to ensure smooth functioning. It has been further argued that there is a dire need for joint training of civilian and military officials in order for them to understand the functioning of other departments better and to reduce the trust deficit. Some civilian officials belonging to civil services also pointed out that they have ‘military attachment’ as an essential part of their common training programme (CTP) and it helps them better understand the Pakistan Army’s functioning. However, the COVID-19 response alone is unlikely to cause any significant change in the challenges faced by the two departments whilst collaborating, but this experience of working together has allowed us to understand the functional dynamics of each department. Recognition of the factors influencing the coordination between the two agencies is of prime importance. Therefore, this article suggests that efforts should be made to reduce challenges and increase cooperation prospects.

## Footnotes

1 Each year, a significant number of members of Shi’ite community from Pakistan visit the holy site of Karbala, where Imam Hussain R.A is buried. As Iran was an epicentre of the pandemic because of inadequate quarantining facilities at the border and negligence of both Iranian and Pakistani authorities, this year many pilgrims caught COVID-19 at the quarantine centre at the border.

2 Ehsaas Emergency Cash programme has been launched by the Prime Minister of Pakistan, Mr. Imran Khan, on 01 April 2020 with regard to the financial difficulty being experienced by the helpless because of coronavirus crisis. The budgetary help under the programme is intended to assist them with purchasing rations. The programme covers approximately 12 million families (Khan 2020; Pakistan Today 2020).

3 For details on ‘Resource management System’ and ‘National Information Technology Board, please visit https://nitb.gov.pk/Detail/YzE1ODI0OTAtMjJmNS00NDVjLTg2YjctYTE1Y2U4Y2NkODdl

4 For further details regarding AFIRM, please visit https://nitb.gov.pk/Detail/YzE1ODI0OTAtMjJmNS00NDVjLTg2YjctYTE1Y2U4Y2NkODdl

5 The 18th Amendment of the constitution of Pakistan enhanced the autonomy of the provinces. Under this amendment, the provinces were made exclusively responsible for healthcare (Mandviwalla 2020).
